# Standardization of Syndrome Differentiation Defined by Traditional Chinese Medicine in Operative Breast Cancer: A Modified Delphi Study

**DOI:** 10.1155/2015/820436

**Published:** 2015-10-01

**Authors:** Qianqian Guo, Qianjun Chen

**Affiliations:** Department of Mammary Disease, Guangdong Provincial Hospital of Chinese Medicine, Guangdong, China

## Abstract

*Objective*. The aim of this study was to establish the standardization of syndrome differentiation of operative breast cancer treated with Traditional Chinese Medicine (TCM) by the modified Delphi method. *Method*. A literature search for standardization of syndrome differentiation of operative breast cancer was conducted and eligible articles were identified in indexed databases from 1982 to 2013. We carried out two rounds of investigation between March and October 2013 and organized 20 experts who focused on TCM or integrative medicine in breast cancer research. Experts' judgments were collected *via* posted questionnaires or e-mail. A final evaluation was carried out after the end of both rounds. *Result*. The response ratio of the 1st round investigation reached 100%, and two experts were excluded due to the uncompleted questionnaire. The 2nd round investigation was completed by 18 experts in the 1st round panel board. In both rounds, the experts agreed that the stage of breast cancer defined by TCM could be divided into the perioperation period, the perichemotherapy period, the periradiotherapy period, and the consolidation period. *Conclusion*. We identified the feasibility and reasonability to establish the standardization of syndrome differentiation of operative breast cancer. According to the suggestions from experts in our Delphi study, we preliminarily established the TCM standard of syndrome differentiation based on different treatment stages of operative breast cancer.

## 1. Introduction

Globally, breast cancer is the most frequently diagnosed cancer and the second cause of cancer death in women. According to the statistics from the World Health Organization in 2011, breast cancer has become a major factor leading to cancer death in women worldwide and accounted for 14% of global cancer deaths [1]. Meanwhile, based on the statistics from the American Cancer Society estimates, there would be 235,030 new cases diagnosed with breast cancer in 2014, and 40,430 cases might die [2]. Clinical investigation found that although breast cancer incidence ratio was significantly increased around the world in the past few years, the survival period was greatly improved due to the distinguished advancements made by the multidisciplinary treatment [3–6].

Long-term clinical practice showed that TCM could reduce the side effects of chemo-drugs and improve the patient's immunity as well. Regarding the same disease, there are varieties of syndromes related to different climates and geographical factors. Currently, the syndrome differentiation defined by TCM on breast cancer treatment is not unified, and the related description or records in the ancient books and literature are limited, resulting in a severe impact on the standardization of TCM treatment in breast cancer. Therefore, it is necessary to explore and establish standard criteria of syndrome differentiation of TCM on breast cancer. Based on the preliminary research of the TCM syndromes, we applied the modified Delphi method to obtain the consensus on the criteria of syndrome differentiation in breast cancer treatment.

The Delphi method is a consensus methodology attempting to assess the extent of agreement and to resolve disagreement in medical and health service research [7, 8]. Its main characteristics include anonymous feedback and statistics. Application of Delphi method in medical research originated from the nursing work in 2000. Mitten-Lewis [9] organized 28 educationalists and 43 nursing experts to form a committee aiming at studying the skill and ability obstacles faced by nurses in Australia. Thereafter the Delphi method was gradually applied in various fields of medicine, such as therapeutic strategy evaluation of esophageal cancer, breast cancer, and colorectal cancer [10]. In the field of Chinese medicine, Delphi can be used in the research of the syndrome distribution, the standard for diagnosis disease, and so on. Here, our study aims to establish the standardization of syndrome differentiation of operative breast cancer treated with TCM by using the modified Delphi method.

## 2. Participants and Methods

### 2.1. Selection Criteria

Delphi methodology suggests that the number of advisory experts can range from ten to fifty. According to the principle of representation and authority, twenty nationwide experts who majored in TCM and integrative medicine were selected in the first round. Based on the first round results, eighteen experts in the first round were further consulted in the second round.

Twenty clinicians working in different regions were selected for evaluating the syndrome results based on their experiences in breast cancer treatment by TCM. In order to increase the credibility of our results, the factor of expert's geographical distribution was also considered. All participating experts were engaged in breast cancer research for at least ten years, and each expert had published at least ten research articles on breast cancer therapy. Their academic position was associate professor or above.

The leading group was formed by key members of the Chinese Medical Society of Breast Diseases Prevention and Control Cooperative Work Committee, who are responsible for the design of the questionnaire. These four professors were the most respected leaders in research and administrative authorities in China. The mean working experience of the leading group was more than 20 years.

### 2.2. Literature Review

A literature review was performed to define the initial list of symptoms of breast cancer. The keywords were “breast cancer in TCM” and “different research of breast cancer in TCM.” The related articles published between 1982 and 2013 from two databases were selected. Case reports and articles based on subjective experience and treatment were excluded.

### 2.3. The Delphi Procedure

To obtain a consensus among experts, a Delphi process was used. The method is a way to obtain expert opinion in a systematic manner. Experts are recruited individually and anonymously. The survey is conducted over several rounds, and the results are analyzed and finally reported to the leading group. The process is considered completed when there is a convergence of opinion or when a point of diminishing returns is reached.

The protocol and consensus assessment used in the Delphi study were based on the structured integration of empirical data and experts' judgments [8]. The Delphi model could systematically evaluate the final agreement based on the individual opinion. According to the literature search, a presurvey questionnaire was suggested to be designed to ensure the accuracy and relevance, and the second round questionnaire was generated based on the results from the first round. We carried out two rounds of investigation between March and October 2013.

### 2.4. Data Collection and Analysis

In each round, experts' judgments were collected* via* post or e-mail using the designed questionnaire. Meanwhile, the questions in both rounds were the same and the feedback of the first round results was also released to the experts during the second round investigation. Each questionnaire included two parts: one part described the experts' opinion on the classification of treatment phase and syndrome differentiation and the other part focused on the definition of each therapeutic phase (see Table 3). The level of agreement of each question was defined as “yes” or “no.” Participants were expected to give their opinion on each question.

The quantitative analysis was carried out by calculating the supportive ratio for each question. It was considered that the experts reached a consensus on a question whenever the supportive ratio exceeds 50%.

## 3. Results

### 3.1. First Round

**Figure 1:**
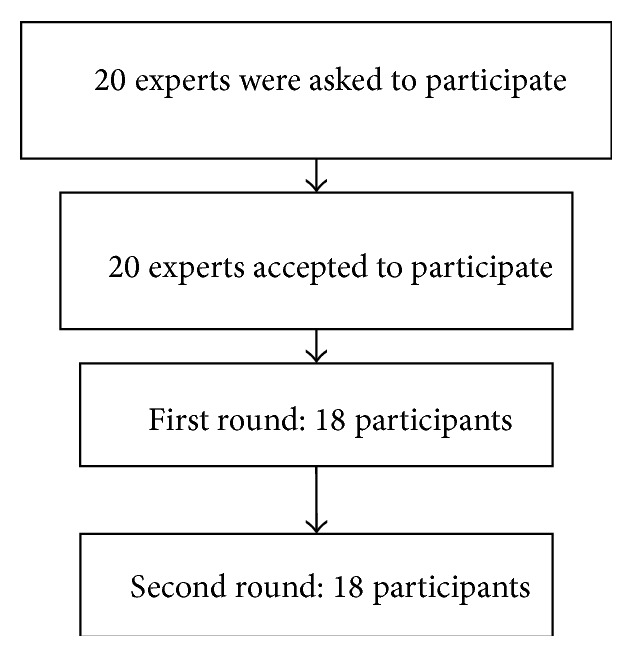
Flow of participants through the Delphi study.

In the first round, the questionnaires were mailed to twenty breast experts and with a positive coefficient and callback rate of 100%, respectively. However, because two questionnaires were unqualified, the effective rate only reached 80%. All experts are associate professors or professors with work experiences over ten years. Eleven experts were with academic experiences more than twenty years and took up 55% of the whole experts (Figure 1), suggesting that the experts have high representation and authority in this study.

18 experts in the first round reached an agreement that the TCM treatment of the operative breast cancer could be divided into four phases including perioperative period, perichemotherapy period, periradiotherapy period, and consolidation period. With regard to syndrome differentiation, only 10 experts agreed that the “sick undocumented type” deems necessary in the perioperative period (55.56%). Besides this, the approval ratio for other syndrome differentiations all reached over 70% (Table 1).

For the definition of each therapeutic phase, the results from the first round investigation were listed as follows: the perioperation refers to the period from surgical admission to the beginning of the first cycle of chemotherapy; the perichemotherapy period is from the start of chemotherapy until one week later of the last cycle of chemotherapy; the periradiotherapy period is from the beginning of the radiotherapy to one week later of the endpoint; finally, the consolidation period refers to the five years after the end of radiotherapy and/or chemotherapy. During the presurvey, all experts reached an agreement about the time length definition on the perioperation and consolidation period, but not for the perichemotherapy and periradiotherapy period. There were three different opinions about the time of the perichemotherapy and the periradiotherapy period. The first opinion considered that the peri-chemotherapy/radiotherapy period refers to the start of chemotherapy/radiotherapy until one week later after the endpoint; the second one claimed that the peri-chemotherapy/radiotherapy period refers to the start of chemotherapy/radiotherapy until two weeks later after the endpoint, and the last one recommended that the periods should be from the start of chemotherapy/radiotherapy until one month later after the endpoint (Table 2).

### 3.2. Second Round

In the second round investigation, eighteen questionnaires were all returned with a recovery ratio of 100%. According to the first round conclusions, breast cancer TCM treatment was divided into the perioperation period, the perichemotherapy period, the periradiotherapy period, and the consolidation period. In addition to the fact that the “sick undocumented type” was revealed with only 55.56% approval ratio, the rest of other syndrome differentiations were approved with more than 80.00% support from experts (Table 2). Therefore, we finally decided to kick out the syndrome “sick undocumented type” from the diagnostic criteria.

With regard to the time length of each TCM therapeutic phase, the second round results were listed in Table 4. Based on the results, we finally decided to determine the period of perichemotherapy and periradiation therapy lasting to two weeks after the end of treatment.

## 4. Discussion

In the past two decades, Delphi study has not only become a widely applied prediction model but also emerged as an important means of assessment and decision-making strategy [11, 12]. Since the method is based on scientific data collection and analysis, it can be used as a powerful tool to evaluate the clinical experience of TCM experts.

The advantages of Delphi depend on the open questionnaire, which can bring brainstorm and mutual discussion, while its disadvantages include the relatively long time, curt feedback, and the timeliness. The classic Delphi method was generally divided into four rounds, but the modified Delphi method cannot include the consultation round. As long as the opinion of experts reached a consensus, the investigation can be terminated and therefore avoid the long time for consultation. During the consultation process, it is better not to adopt “a piece of paper law” in the first round in order to refrain from disperse answers. In this study, according to the modified Delphi method, a predesigned questionnaire was designed based on the preliminary literature search results, which is equivalent to the completion of the first round consultation and effectively shortens the consultation period. Meanwhile, the questionnaire design should have as much detail as possible and leave enough space for expert review.

On the other hand, the selection of experts is also a critical factor determining the final successful results [13]. The number of experts was generally recommended between 10 and 50 people and must be from the same academic field. The study relies on the Surgery Branch of Chinese Medical Association Professional Committee of Breast Disease Platform and has established good cooperative relations in the process of preliminary enquiry. Regarding distributing the questionnaire, we contacted experts by a combination of post, e-mail, and telephone to explain the questionnaire and listen to their suggestions or recommendations. In fact, our result demonstrated that above communications effectively enhance the enthusiasm of experts and made up the flaws of tight time and curt answers.

In previous studies involving the use of Delphi method for the syndrome differentiation defined by TCM in breast cancer, we found that the inconsistence of experts in each round investigation would greatly impact the final statistical results. In order to avoid such mistake, our study guaranteed that the experts in both rounds had high consistency by selecting eighteen experts from the first round investigation. Meanwhile, we followed the principle of the Delphi method to ensure that the consultation and the leading group consisted of different people in order to achieve the high authenticity and accuracy of the results.

Previous research suggested that the perichemotherapy/radiotherapy refers to the period from the start until one week later after the end of chemotherapy or radiotherapy. However, our study indicated that experts tended to define the period extending two weeks later after the end of treatment. Meanwhile, the experts pointed out that the side effects of chemotherapy/radiotherapy could be generally relieved in two weeks after the end of treatment in the presurvey; therefore they proposed to define the above-mentioned time length and the suggestion was also supported by most experts in consultation. What is more, in clinical setting we also recommended that patients start to receive endocrine therapy two weeks later after the end of chemo/radiotherapy, since the side effects will be usually relieved at that time point. Based on clinical observation and experts' discussion, the perichemotherapy/radiotherapy was finally defined two weeks later after the end of treatment.

At present, the treatment of breast cancer in western medicine includes surgery, chemotherapy, radiotherapy, endocrine therapy, and molecular targeted therapy and has developed rapidly, but the side effects of western medicine severely influenced patients' quality of life and prognosis. Therefore, TCM plays an important role in relieving the side effects brought by chemotherapy or radiotherapy. The aim of the syndrome differentiation defined by Traditional Chinese Medicine in operative breast cancer is to treat the patients on different Zheng composition and to get a better quality of life.

The establishment of each syndrome differentiation involved in this study was based on systematic literature review and experts' discussion. However, some of the methods and conclusions may be premature and require further research to consummate the detailed syndrome classification in order to achieve higher applied significance in clinical practice.

## Figures and Tables

**Table 1:** The experts' opinion on the classification of treatment phase and syndrome differentiation in the first round.

Stage	Syndrome manifestations	Number	Percent
The operation period			
Preoperation	Stagnation of liver-qi and phlegm	18	1
	Phlegm and blood stasis syndrome	18	1
	Chongren imbalance	18	1
	Deficiency of healthy energy and sthenia of evil	18	1
	Sick undocumented type	10	0.56
Postoperation	Spleen disharmony syndrome	18	1
	Deficiency of vital energy and blood pattern	18	1
	Qi-Yin deficiency type	16	0.89
Chemotherapy period	Spleen disharmony syndrome	18	1
	Deficiency of vital energy and blood pattern	16	0.89
	Qi-Yin deficiency type	15	0.83
	Deficiency of the liver and kidney	15	0.83
	Deficiency of the spleen and kidney	17	0.94
Radiotherapy period	Deficiency of vital energy and blood pattern	14	0.78
	Qi-Yin deficiency type	17	0.94
	Yin-chun deficiency	17	0.94
	Deficient yin induces vigorous fire	13	0.72
Consolidation period	Deficiency of vital energy and blood pattern	17	0.94
	Qi-Yin deficiency type	17	0.94
	Deficiency of the spleen and kidney	17	0.94
	Chongren imbalance	14	0.78

**Table 2:** The experts' opinions on the time length of perichemotherapy/radiotherapy in the first round investigation.

Proposal	Opinion
Extended to two weeks	55.56%
Extended to one week	27.78%
Extended to one month	16.67%

**Table 3:** The experts' opinion on the classification of treatment phase and syndrome differentiation in the second round.

Stage	Syndrome manifestations	Number	Percent
The operation period			
Preoperation	Stagnation of liver-qi and phlegm	7	1
	Phlegm and blood stasis syndrome	18	1
	Chongren imbalance	18	1
	Deficiency of healthy energy and sthenia of evil	17	0.94
	Sick undocumented type	10	0.56
Postoperation	Spleen disharmony syndrome	17	0.94
	Deficiency of vital energy and blood pattern	18	1
	Qi-Yin deficiency type	15	0.83
Chemotherapy period	Spleen disharmony syndrome	18	1
	Deficiency of vital energy and blood pattern	17	0.94
	Qi-Yin deficiency type	16	0.89
	Deficiency of the liver and kidney	17	0.94
	Deficiency of the spleen and kidney	18	1
Radiotherapy period	Deficiency of vital energy and blood pattern	15	0.83
	Qi-Yin deficiency type	18	1
	Yin-chun deficiency	18	1
	Deficient yin induces vigorous fire	15	0.83
Consolidation period	Deficiency of vital energy and blood pattern	18	1
	Qi-Yin deficiency type	18	1
	Deficiency of the spleen and kidney	18	1
	Chongren imbalance	15	0.83

**Table 4:** The experts' opinions on the time length of perichemotherapy/radiotherapy in the second round investigation.

Proposal	Opinion
Extended to two weeks	72.22%
Extended to one week	22.22%
Extended to one month	5.56%
